# Investigation of Analgesic, Anti-Inflammatory, and Thrombolytic Effects of Methanolic Extract and Its Fractions of *Dischidia bengalensis*: In Vitro and In Vivo Studies with In Silico Interventions

**DOI:** 10.3390/molecules30183724

**Published:** 2025-09-12

**Authors:** Ainun Nahar, Md. Jahin Khandakar, Md. Jahirul Islam Mamun, Md. Hossain Rasel, Abu Bin Ihsan, Asef Raj, Saika Ahmed, Mohammed Kamrul Hossain, Md Riasat Hasan, Takashi Saito

**Affiliations:** 1 Department of Pharmacy, Faculty of Biological Sciences, University of Chittagong, Chittagong 4331, Bangladesh; ainun.pharmacy@easternuni.edu.bd (A.N.); jahirulmamun3@gmail.com (M.J.I.M.); msdrasel422@gmail.com (M.H.R.); mkhossain73@yahoo.com (M.K.H.); 2 Department of Pharmacy, Faculty of Life Science, Eastern University, Dhaka 1345, Bangladesh; 3 Department of Oceanography, Faculty of Marine Science and Fisheries, University of Chittagong, Chittagong 4331, Bangladesh; khandakarjahin@gmail.com; 4 School of Pharmacy, BRAC University, Dhaka 1212, Bangladesh; asef.raj@bracu.ac.bd; 5 Department of Chemistry, University of Dhaka, Dhaka 1000, Bangladesh; saika@du.ac.bd; 6 Division of Clinical Cariology and Endodontology, Department of Oral Rehabilitation, School of Dentistry, Health Sciences University of Hokkaido, Tobetsu 061-0293, Hokkaido, Japan

**Keywords:** *Dischidia bengalensis*, analgesic, anti-inflammatory, thrombolytic, GC-MS, molecular docking

## Abstract

In a continued search for novel plant-based therapeutics with multi-target pharmacological potential, the medicinal plant *Dischidia bengalensis* (Apocynaceae) was investigated for the first time for its anti-inflammatory, analgesic, and thrombolytic properties, addressing critical therapeutic areas such as rheumatoid arthritis, acute pain, and thrombosis. The methanolic extract and solvent fractions (dichloromethane, n-hexane, and ethyl acetate) were evaluated through integrated in vivo, in vitro, and in silico approaches. Phytochemical screening and GC–MS profiling revealed a diverse array of bioactive constituents, including fatty acids, terpenoids, and phenolic derivatives, many of which are reported to exhibit pharmacological activities. In vivo assays demonstrated that the methanolic extract (400 mg/kg) markedly suppressed carrageenan-induced paw edema (92.31% inhibition) from the 2nd to 4th hour (*p*  <  0.05, *p*  <  0.01), while the n-hexane fraction produced the most pronounced analgesic response in both writhing and tail-immersion models (*p*  <  0.001). Furthermore, the methanolic extract displayed significant thrombolytic activity (33.38  ±  4.27% at 20 mg/mL, *p* < 0.001) in human blood clot lysis, suggesting potential application in cardiovascular disorders. The scientific novelty of this study was further underscored by in silico molecular docking, ADME/T, and PASS prediction studies. Key bioactive compounds, identified by GC-MS, showed strong binding affinities and promising drug-like properties against pivotal human targets such as TNF-α (PDB: 2AZ5), COX-2 (PDB: 6COX), and tissue plasminogen activator. These findings conclusively establish *D. Bengalensis* as a promising and novel source of lead compounds for the development of novel therapeutics against inflammatory, pain-related, and cardiovascular disorders.

## 1. Introduction

Medicinal plants have long provided valuable bioactive compounds for the treatment of human diseases, offering therapeutic benefits with often fewer side effects than synthetic drugs [1]. Within this context, the genus *Dischidia* (family Apocynaceae) comprises several species traditionally used in South and Southeast Asia to treat inflammation, skin infections, respiratory ailments, pain, and gastrointestinal disorders. Phytochemical investigations have so far identified 21 bioactive compounds from four species -*D. nummularia*, *D. alboflava*, *D. bengalensis*, and *D. formosana*, which exhibit at least 18 distinct pharmacological activities, highlighting the genus as a promising source of therapeutic agents [2]. Among these, *Dischidia bengalensis* Colebr., an epiphytic climbing plant, has been valued in folk medicine but remains scientifically underexplored. For instance, the Khasia tribe of Sylhet, Bangladesh, traditionally uses the leaf and stem juice of *D. bengalensis* to improve hearing and to relieve menstrual and gastric pain [3,4]. Despite such ethnomedicinal claims, no systematic studies have yet evaluated its anti-inflammatory, analgesic, or thrombolytic properties, highlighting an important research gap.

The relevance of studying *D. bengalensis* is underscored by the limitations of current therapies for inflammation, pain, and thrombotic diseases. Inflammation, while essential as a protective physiological response, contributes to chronic disorders such as arthritis, cardiovascular disease, and neurodegenerative conditions when excessive or persistent [5,6]. Conventional anti-inflammatory agents, including NSAIDs and corticosteroids, are effective but associated with adverse effects such as renal impairment and gastrointestinal toxicity [7]. These limitations have prompted the search for safer alternatives, including natural products [8]. Contemporary research methodologies, such as molecular studies, in vitro assays, and in vivo models, etc., facilitate both mechanistic insights and the identification of novel anti-inflammatory leads [9].

Similarly, pain is one of the most prevalent and debilitating health problems worldwide, significantly affecting quality of life and placing a heavy burden on healthcare systems [10]. Although opioids and NSAIDs remain the mainstay of analgesic therapy, they are associated with tolerance, dependence, and gastrointestinal complications [11,12]. New analgesic drugs are being explored as alternatives to NSAIDs and opiates, with growing focus on plant-based medicines due to their low cost, fewer side effects, and continued reliance by about 80% of the global population, as noted by the World Health Organization (WHO) [13,14].

Thrombotic diseases, including myocardial infarction and stroke, are also major causes of global mortality [15], resulting from blood clots that obstruct circulation and cause tissue damage [16]. Thrombolytic agents like tissue plasminogen activator (tPA) are indispensable in clinical practice but are limited by severe side effects, including hemorrhage [17]. Standardized models such as fibrin plate assays and in vitro clot lysis tests are commonly used to screen new agents with improved safety profiles [18]. In this context, medicinal plants represent attractive sources of novel thrombolytic compounds [19].

In addition, in silico tools such as molecular docking and virtual screening have become integral to modern drug discovery, enabling rapid identification and optimization of lead compounds while saving time and resources before laboratory testing [20,21,22].

Taken together, the traditional use of *D. bengalensis*, its phytochemical potential, and the urgent need for safer anti-inflammatory, analgesic, and thrombolytic agents provide a strong rationale for this study. To the best of our knowledge, this is the first investigation of the phytochemical composition and pharmacological effects of *D. bengalensis*. By bridging ethnomedicinal knowledge with scientific validation, this work highlights the potential of *D. bengalensis* as a source of novel therapeutic agents for pain, inflammation, and cardiovascular disorders.

## 2. Results

### 2.1. Phytochemical Screening

The phytochemical analysis of the methanolic extract of *D. bengalensis* is displayed in Table 1.

### 2.2. GC-MS Profiling

GC-MS (gas chromatography-mass spectrometry) analysis was conducted to identify the compounds present in the methanolic extract of *D. bengalensis*. The most abundant compound was n-Hexadecanoic acid (12.99%), followed by 9-Octadecenamide, (*Z*)-(8.58%). The GC-MS chromatogram is presented in Figure S1 of the Supplementary Materials, with the identified compounds detailed in Table 2.

### 2.3. Acute Toxicity Evaluation and Dose Determination

No death and abnormalities such as restlessness, convulsions, reduced motor activity, diarrhea, coma, and lacrimation occurred in the test animals at the experimental dosages for each of the groups. Therefore, LD_50_ was determined to be higher than 4 g/kg of body weight. To be in line with previous in vivo studies using plant extracts [23,24] and safety limit derived from acute toxicity evaluation, 200 mg/kg as a moderately effective dose, and 400 mg/kg as a higher dose were chosen to assess dose-dependence.

### 2.4. Anti-Inflammatory Activity

#### Carrageenan-Induced Paw Edema

The anti-inflammatory activity of *Dischidia bengalensis* extracts was assessed using the carrageenan-induced paw edema model in mice. The standard drug (indomethacin) showed significant inhibition of paw edema, with a maximum inhibition of 96.15% at the 4th hour.

Among the extracts, the methanolic extract at 400 mg/kg (ME-400) exhibited the highest anti-inflammatory effect, showing 92.31% inhibition from the 2nd (*p* < 0.05) to 4th hour (*p* < 0.01), closely approaching the standard. The ME-200 group also showed strong activity, reaching 92.31% inhibition at the 4th hour, though slightly delayed in onset. Other fractions demonstrated notable anti-inflammatory effects in a dose-dependent manner. Elevation of the dose from 200 to 400 mg/kg produced a more pronounced biological effect. The NH and DCM fractions demonstrated significant activity (up to 88.46%; *p* < 0.001) at various doses. The EA fraction showed moderate effects at 400 mg/kg but was ineffective at 200 mg/kg. These findings highlight the potential of the methanol extract and its fractions as anti-inflammatory agents (Table 3).

### 2.5. Analgesic Activity

#### 2.5.1. Acetic Acid-Induced Writhing Method

The present study demonstrated a dose-dependent relationship between the tested doses of 200 mg/kg and 400 mg/kg. The analgesic effect significantly increased when the dose was raised from 200 mg/kg to 400 mg/kg, indicating that higher doses elicited a stronger biological response. The standard drug, diclofenac (10 mg/kg), significantly reduced the number of writhes (5.6 ± 0.24), resulting in 88.06% inhibition, confirming assay validity. Among the extracts, the methanolic extract at 400 mg/kg (ME-400) exhibited the most potent analgesic effect (7.8 ± 0.2 writhes; 83.37% inhibition), closely approaching the standard. ME-200 also showed significant inhibition (61.83%), indicating a dose-dependent effect. The n-hexane extract (NH-400) showed 80.60% inhibition, while NH-200 was moderately effective (38.81%). The ethyl acetate extract (EA-400) showed strong activity (74.84% inhibition), outperforming its 200 mg/kg counterpart (65.46%). The dichloromethane fraction (DCM-400) exhibited moderate analgesic activity (54.16% inhibition), whereas DCM-200 was the least effective among all groups (17.48% inhibition).

Overall, the ME-400 and NH-400 extracts demonstrated the highest analgesic effects, comparable to the standard, while the EA and DCM extracts showed moderate to significant dose-dependent activity (Table 4).

#### 2.5.2. Tail Immersion Test

The central analgesic activity of *Dischidia bengalensis* extracts was evaluated using the tail immersion test, with pentazocine (10 mg/kg, p.o.) as the reference standard. Reaction times (in seconds) and corresponding % Maximum Possible Effect (%MPE) and % elongation of latency were measured at 30, 60, 90, and 120 min post-administration. All fractions exhibited analgesic activity in an approximately dose-dependent manner, although the variation in effect between doses was not substantial across all fractions.

The standard drug exhibited significantly increased latency (%MPE: 59.19% at 90 min) and consistent elongation of latency time, peaking at 62.20% at 90 min. Among the tested extracts, the methanolic extract (ME) showed the most potent and sustained central analgesic activity. ME-200 demonstrated the highest efficacy, reaching 9.84 ± 1.17 s (reaction time) and 56.64% MPE at 60 min, with corresponding 65.01% elongation, which exceeded the standard at that time point. ME-400 also produced robust analgesia (up to 54.21% MPE and 59.09% elongation at 120 min). The n-hexane fraction (NH) showed strong effects as well, with NH-400 achieving 53.56% MPE and 58.36–58.89% elongation across 90–120 min. Similarly, ethyl acetate extracts (EA-400 and EA-200) showed notable results, maintaining 50.44–48.76% MPE and elongation above 55% through most time points. The dichloromethane fraction (DCM-400) showed moderate but consistent analgesic activity, peaking at 49.37% MPE and 57.22% elongation at 120 min.

All test groups showed statistically significant (*p* < 0.001) increases in tail withdrawal latency compared to the control. The methanol and n-hexane extracts exhibited centrally mediated analgesic effects comparable to pentazocine, indicating the presence of active phytoconstituents with opioid-like or CNS-modulatory potential (Table 5 and Table 6).

### 2.6. Thrombolytic Activity

#### Human Blood Clot Lysis Method

**Figure 1:**
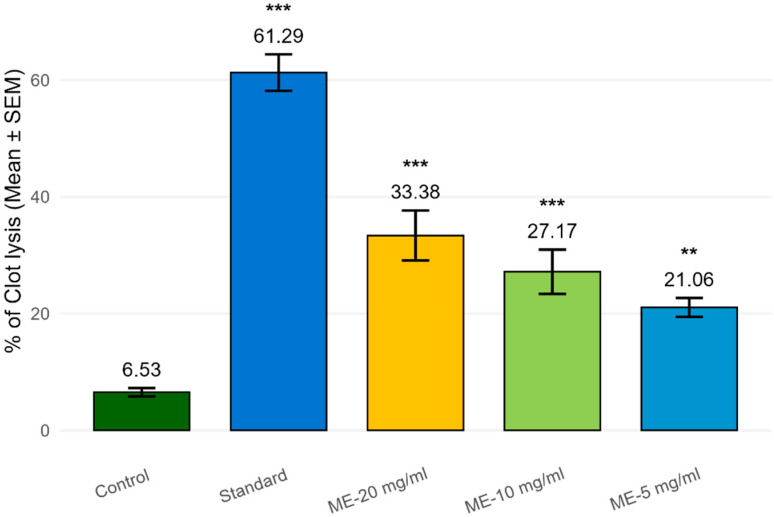
Clot lysis of blood samples of normal subjects by different concentrations of methanol extract. Values are expressed as mean ± SEM (*n* = 10). Statistical significance was assessed vs. negative control (** *p* < 0.01, *** *p* < 0.001).

The thrombolytic potential of the methanolic extract (ME) was evaluated at concentrations of 5, 10, and 20 mg/mL, and compared against the standard thrombolytic agent and negative control following a standardized protocol described in the Section 4. Due to a limited quantity of the other fractions, the thrombolytic activity assay was prioritized for the methanolic extract, which demonstrated the highest yield and broadest phytochemical profile in preliminary tests. A clear concentration-dependent thrombolytic response was observed. The effect became more prominent as the concentration of the tested sample increased. The standard (streptokinase) exhibited a significant clot lysis of 61.29 ± 3.12% (*p* < 0.001), whereas the negative control (distilled water or saline) showed minimal clot dissolution (6.53 ± 0.71%), confirming the validity of the assay. The methanol extract demonstrated a dose-dependent clot lysis effect with percentages of 33.38% (*p* < 0.001), 27.17% (*p* < 0.001), and 21.06% (*p* < 0.01) at concentrations of 20 mg/mL, 10 mg/mL, and 5 mg/mL, respectively, compared to the control (normal saline). The methanol extract at 20 mg/mL showed the highest thrombolytic activity among the different concentrations of the tested sample. Although the ME did not reach the clot lysis level of the standard, the results indicate significant thrombolytic activity, suggesting the presence of bioactive phytoconstituents with fibrinolytic or plasmin-activating properties. The extract’s activity at higher concentrations reached over 54% of the standard, indicating a moderately strong thrombolytic potential (Figure 1).

### 2.7. In Silico Study

#### 2.7.1. ADME/T Analysis

The pharmacokinetics and drug-likeness of the phytochemicals were assessed to determine their therapeutic potential before docking analysis. All substances met Lipinski’s rule of five, and the pharmacokinetic parameters presented in Table 7 show no mutagenic or carcinogenic effects. All of the compounds in ME exhibited no toxicity, and in silico analyses using pKCSM and Swiss ADME verified pharmacological similarity.

#### 2.7.2. Molecular Docking

**Figure 2:**
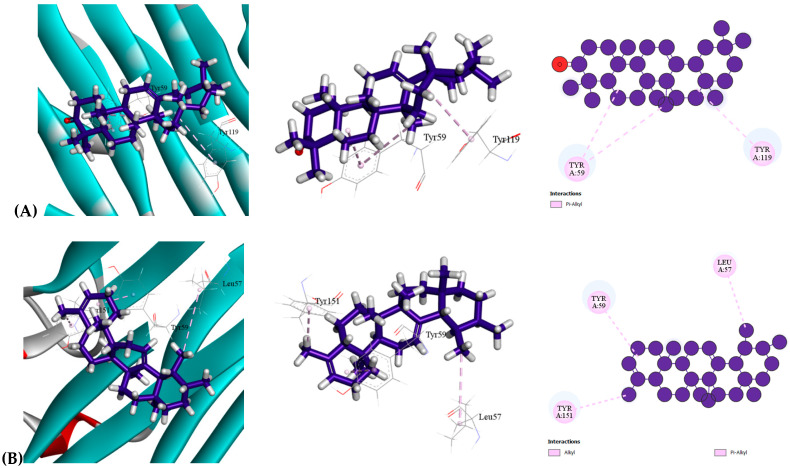

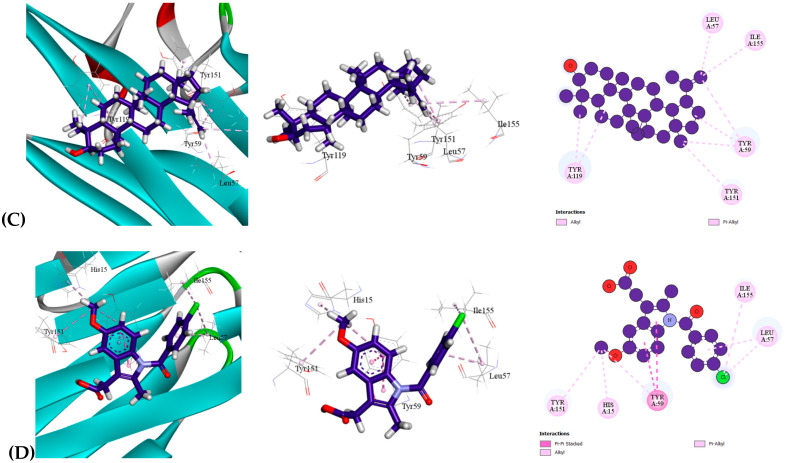
Molecular docking interaction of compounds against the TNF-alpha (PDB: 2AZ5): (**A**) beta-Amyrone, (**B**) 24-Norursa-3,12-diene, (**C**) Epilupeol, (**D**) Indomethacin (Standard).

**Figure 3:**
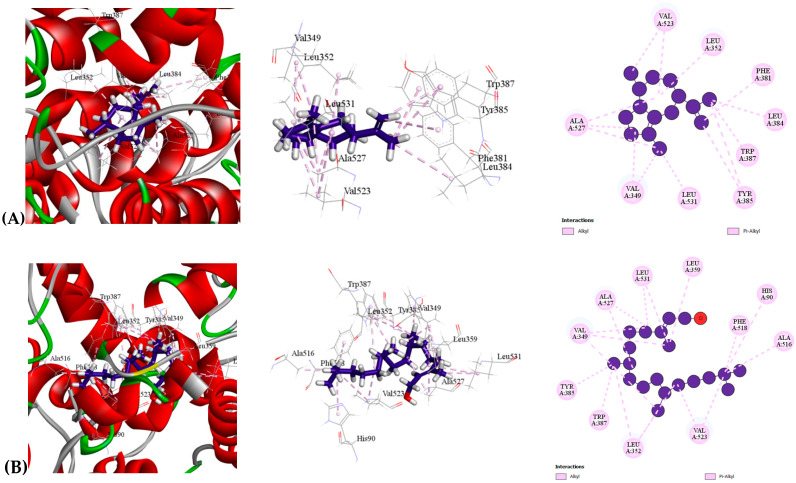

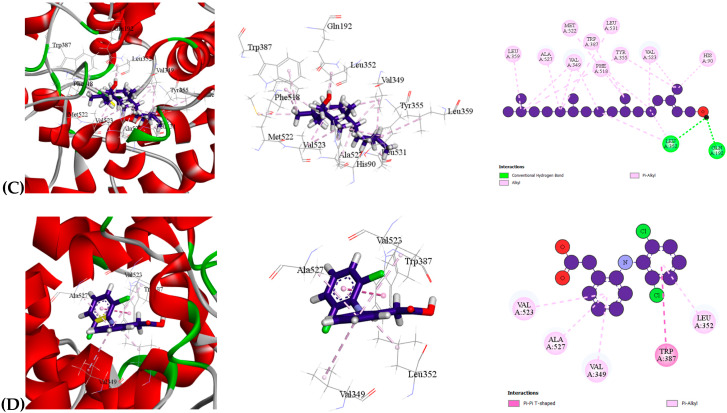
Molecular docking interaction of compounds against the human cyclooxygenase-2 inhibitor (PDB: 6COX): (**A**) Aciphyllene, (**B**) 3,7,11,15-Tetramethyl-2-hexadecen-1-ol, (**C**) Phytol, (**D**) Diclofenac (Standard).

**Figure 4:**
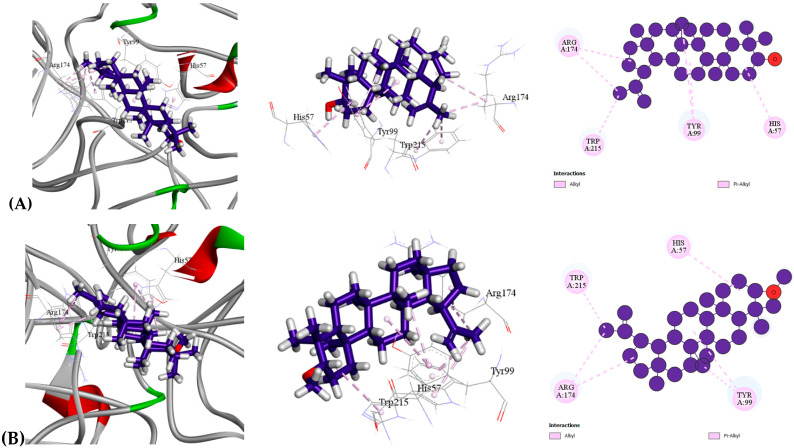

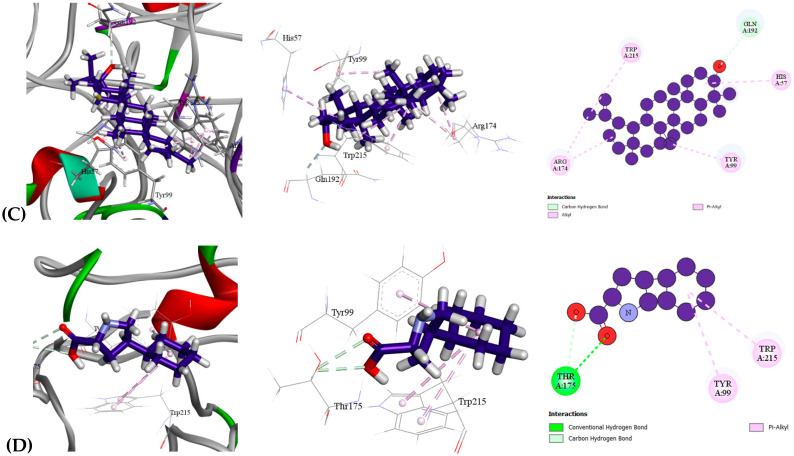
Molecular docking interaction of compounds against the human tissue plasminogen activator, tPA (PDB: 1A5H): (**A**) Epilupeol, (**B**) Lupeol, methyl ether, (**C**) Lupeol, (**D**) Estreptoquinasa (Standard).

The combined docking score for anti-inflammatory, analgesic, and thrombolytic action is displayed in Table 8 and Table 9 and Figure 2, Figure 3 and Figure 4 display the docking scores and interaction assessments of the top three ME compounds as well as the reference medication.

##### Molecular Docking for Anti-Inflammatory Activity

The anti-inflammatory efficacy of the selected bioactive compounds from MEDS was evaluated by analyzing their interactions with TNF-alpha (PDB: 2AZ5). Each compound demonstrated significant binding affinity to the target receptor. Among them, beta-Amyrone exhibited the highest binding affinity (−7.3 kcal/mol), followed by 24-Norursa-3,12-diene (−7.1 kcal/mol) and Epilupeol (−6.8 kcal/mol). Notably, most of the selected compounds showed higher binding affinities than the conventional drug Indomethacin (−6.3 kcal/mol). This study revealed that beta-Amyrone shares a similar binding mechanism and interaction pattern with the standard drug indomethacin [Table 9 (Section 1), Figure 2]. The docking analysis indicated that beta-Amyrone formed three Pi-Alkyl bonds with TYR59 (2) and TYR119 at short intermolecular distances, suggesting strong binding to the active site of TNF-alpha. Given that hydrophobic interactions primarily drive drug-receptor binding, shorter bond lengths (<5 Å) contribute to stronger binding affinities and higher docking scores. These findings highlight the potential of beta-Amyrone as a promising anti-inflammatory agent.

##### Molecular Docking for Analgesic Activity

Regarding their analgesic properties, all compounds demonstrated affinity for the human cyclooxygenase-2 inhibitor (PDB: 6COX). Aciphyllene exhibited the highest binding affinity (−8.6 kcal/mol), surpassing even the reference drug Diclofenac (−8.4 kcal/mol) [Table 9 (Section 2), Figure 3]. The next most potent compounds were 3,7,11,15-Tetramethyl-2-hexadecen-1-ol (−7.2 kcal/mol) and Phytol (−7.2 kcal/mol). A detailed docking analysis revealed that Aciphyllene formed ten Alkyl bonds at short intermolecular distances with residues VAL349 (2), LEU352, VAL523 (2), ALA527 (3), LEU531, and LEU384, along with four Pi-Alkyl bonds involving PHE381, TYR385 (2), and TRP387. These interactions indicate that Aciphyllene binds strongly to the active site of the human cyclooxygenase-2 inhibitor, underscoring its high affinity and potential as a potent analgesic agent.

##### Molecular Docking for Thrombolytic Activity

In terms of thrombolytic traits, all compounds displayed high affinity for the human tissue-type plasminogen activator (tPA, PDB: 1A5H). Among them, Epilupeol exhibited the highest binding affinity (−9.6 kcal/mol), surpassing even the reference drug Estreptoquinasa (−6.5 kcal/mol) [Table 9 (Section 3), Figure 4]. The next most potent compounds were Lupeol, methyl ether, and Lupeol, all showing binding affinities of −9.5 kcal/mol. A detailed docking analysis revealed that Epilupeol formed two Alkyl bonds at short intermolecular distances with residue ARG174 (2), along with five Pi-Alkyl bonds involving HIS57, TYR99 (2), and TRP215 (2). These interactions indicate that Epilupeol binds strongly to the active site of the human tissue-type plasminogen activator, highlighting its high affinity and potential as a potent thrombolytic agent.

**Table 8:** Docking score of the selected compounds identified from the ME against the respective receptors.

Compounds	PubChem ID	Docking Score (kcal/mol)		
		Anti-Inflammatory (2az5)	Analgesic (6cox)	Thrombolytic (1a5h)
Phenol, 3,5-bis(1,1-dimethylethyl)-	70825	−5.4	−6.4	−6.3
Pentadecanoic acid, methyl ester	23518	−4.1	−6.4	−5.6
Neophytadiene	10446	−4.4	−6.9	−6.5
3,7,11,15-Tetramethyl-2-hexadecen-1-ol	5366244	−4.3	−7.2	−6.7
Hexadecanoic acid, methyl ester	8181	−3.9	−6.4	−5.4
n-Hexadecanoic acid	985	−4.3	−6.4	−6
9,12-Octadecadienoic acid (*Z*,*Z*)-, methyl ester	5284421	−4.4	−6.9	−6.1
8,11,14-Docosatrienoic acid, methyl ester	5364473	−4.2	−7.1	−6.1
11-Octadecenoic acid, methyl ester	5364432	−4.4	−6.6	−5.6
Phytol	5280435	−4.6	−7.2	−6.7
10*E*,12*Z*-Octadecadienoic acid	5282800	−4.3	−7	−6
9,11-Octadecadienoic acid, methyl ester, (*E*,*E*)-	5365686	−3.9	−6.9	−6
9-Octadecenamide, (*Z*)-	5283387	−4.1	−6.8	−5.5
Aciphyllene	565709	−5.9	−8.6	−6.4
Lupeol	259846	−6.8	4.2	−9.5
9,19-Cyclolanost-25-ene-3,24-diol	11419367	−6.2	−1.8	−7.7
Lup-20(29)-en-3-ol, acetate, (3beta)-	92157	−6.3	6	−9.5
Epilupeol	5270628	−6.8	5.2	−9.6
Lupeol, methyl ether	15226333	−6.7	6.1	−9.5
beta-Amyrone	12306160	−7.3	−1.1	−8.4
Stigmasterol	5280794	−6.6	−5.9	−7.9
gamma-Sitosterol	457801	−6.4	−6.4	−7.8
24-Norursa-3,12-diene	91735342	−7.1	4.1	−7.4
Standards (Indomethacin, Diclofenac, Estreptoquinasa)		−6.3	−8.4	−6.5

**Table 9:** In silicobinding affinity and non-bonding interactions of selected compounds from ME for anti-inflammatory (2az5), analgesic (6cox), and thrombolytic (1a5h) activities.

Section Number	Receptor	Compounds Name	Binding Affinity (kcal/mol)	Bond Type	Amino Acids
1	2az5	beta-Amyrone	−7.3	Pi-Alkyl	TYR59 (2), TYR119
		24-Norursa-3,12-diene	−7.1	Alkyl	LEU57
				Pi-Alkyl	TYR59, TYR151
		Epilupeol	−6.8	Alkyl	LEU57, ILE155
				Pi-Alkyl	TYR59 (3), TYR119 (2), TYR151
		Indomethacin (Standard)	−6.3	Pi-Pi Stacked	TYR59 (2)
				Alkyl	LEU57, ILE155
				Pi-Alkyl	HIS15, TYR59, TYR151, LEU57
2	6cox	Aciphyllene	−8.6	Alkyl	VAL349 (2), LEU352, VAL523 (2), ALA527 (3), LEU531, LEU384
				Pi-Alkyl	PHE381, TYR385 (2), TRP387
		3,7,11,15-Tetramethyl-2-hexadecen-1-ol	−7.2	Alkyl	VAL349 (3), ALA516, VAL523 (2), ALA527 (2), LEU352 (3), LEU531 (2), LEU359
				Pi-Alkyl	HIS90, TYR385, TRP387, PHE518
		Phytol	−7.2	Conventional Hydrogen Bond	GLN192, LEU352
				Alkyl	VAL349 (3), VAL523 (2), ALA527, MET522, LEU352 (2), LEU531, LEU359
				Pi-Alkyl	HIS90, TYR355 (2), TRP387, PHE518 (2)
		Diclofenac (Standard)	−8.4	Pi-Pi T-shaped	TRP387, VAL349, VAL523, ALA527, LEU352
3	1a5h	Epilupeol	−9.6	Alkyl	ARG174 (2)
				Pi-Alkyl	HIS57, TYR99 (2), TRP215 (2)
		Lupeol, methyl ether	−9.5	Alkyl	ARG174 (2)
				Pi-Alkyl	HIS57, TYR99 (2), TRP215 (2)
		Lupeol	−9.5	Carbon Hydrogen Bond	GLN192
				Alkyl	ARG174 (2)
				Pi-Alkyl	HIS57, TYR99, TRP215 (2)
		Estreptoquinasa (Standard)	−6.5	Conventional Hydrogen Bond	THR175
				Carbon Hydrogen Bond	THR175 (2)
				Pi-Alkyl	TYR99, TRP215 (2)

### 2.8. PASS Prediction

Eleven chosen compounds of ME were examined for their anti-inflammatory and analgesic properties using the PASS (prediction of activity spectra for substances) online program. Results showed that compounds with Pa higher than Pi have significant molecular potency Table 10.

## 3. Discussion

Phytochemical screening revealed the presence of diverse secondary metabolites such as alkaloids, carbohydrates, flavonoids, tannins, terpenoids, saponins, phenols, and glycosides, etc. These findings suggest the potential of *D. bengalensis* as a source of bioactive compounds with therapeutic properties. Many of these phytoconstituents, such as alkaloids, flavonoids, and terpenoids, contribute to the treatment of various diseases [25]. Many researchers have highlighted the medicinal properties of these compounds. For instance, alkaloids are known for their antipyretic, analgesic, and anti-inflammatory effects [26]. Tannins exhibit astringent properties and are utilized for their therapeutic benefits, including antioxidant, hypoglycemic, and anticancer activities [27]. Triterpenes, another class of compounds identified, are recognized for their anti-inflammatory and anti-tumor properties [28].

The carrageenan-induced paw edema test is widely used to evaluate the efficacy of both steroidal and non-steroidal anti-inflammatory drugs, as it involves multiple inflammatory mediators [29]. This method is also frequently employed to assess the anti-edematous effects of natural products [30]. Carrageenan-induced inflammation is biphasic and time-dependent. The early phase (0–1 h post-injection) involves mediators such as histamine, serotonin, and bradykinin, while the later phase (after 1 h) is mediated by prostaglandins, lysosomal enzymes, and proteases [31,32]. Injection of carrageenan, a polysaccharide, induces paw swelling (edema) in animal models [33]. The edema development triggered by carrageenan mirrors the acute inflammation, orchestrated by histamine, bradykinin, and prostaglandins synthesized via the action of cyclooxygenase. At high doses, the crude methanolic extract of *D. bengalensis* rapidly reduces inflammation, while other fractions exhibit a slower but similar effect. The crude extract effectively inhibits carrageenan-induced paw edema in mice, likely due to the presence of secondary metabolites such as alkaloid, tannin, saponin, terpenoids, flavonoids, and phenolicsetcin the tested extract [34,35,36]. In addition, GC-MS analysis of the plant extract identified a range of bioactive compounds, including phenol, 3,5-bis(1,1-dimethylethyl)-, pentadecanoic acid, methyl ester, n-hexadecanoic acid, 9,12-octadecadienoic acid, methyl ester, 8,11,14-docosatrienoic acid, methyl ester, 11-octadecenoic acid, methyl ester, 10*E*,12*Z*-octadecadienoic acid, 9,11-octadecadienoic acid, methyl ester, 9-octadecenamide, aciphyllene, lupeol, 9,19-cyclolanost-25-ene-3,24-diol, 1-phenanthrenecarboxylic acid derivative, lup-20(29)-en-3-ol acetate, epilupeol methyl ether, stigmasterol, sesquiterpene aldehyde, γ-sitosterol, and 24-norursa-3,12-diene. These phytoconstituents have been previously reported to exhibit significant anti-inflammatory activity, thereby supporting the pharmacological potential of the extract [37,38]. These bioactive compounds may exert their anti-inflammatory effects by inhibiting pro-inflammatory enzymes like cyclooxygenase (COX) and lipoxygenase (LOX) or by promoting anti-inflammatory cytokines such as IL-10 and TGF-β [39]. Most clinically approved anti-inflammatory drugs, such as NSAIDs (e.g., indomethacin, phenylbutazone, aspirin, diclofenac sodium, piroxicam) and hydrocortisone, etc., effectively target the second phase. Based on its performance, the extract demonstrates potent anti-inflammatory activity comparable to that of indomethacin, a standard drug, which acts as a cyclooxygenase inhibitor and markedly reduces paw edema by blocking the release of serotonin, histamine, bradykinin, prostaglandins, and lysozyme enzymes. Therefore, it may be concluded that the anti-inflammatory effect of methanol extract and its different solvent fractions is due to the suppression of protease, lysozymes, histamine, serotonin, bradykinin, and prostaglandins synthesis or activity.

The acetic acid-induced writhing test is a widely used method to evaluate peripheral analgesic efficacy [40]. Pain induction by acetic acid occurs through an indirect mechanism involving the elevation of prostaglandin (PG2 and PG2α) levels at receptor sites within organ cavities, suggesting that acetic acid enhances the release of endogenous mediators [41,42]. Acetic acid induces writhing in experimental animals by activating chemosensitive nociceptors [43]. Nonsteroidal anti-inflammatory drugs (NSAIDs) alleviate this pain by inhibiting sensory neuron activation triggered by inflammatory mediators [44]. The percentage reduction in the number of writhes serves as an indicator of analgesic efficacy [45]. In this study, the n-hexane fraction at both 200 and 400 mg/kg significantly reduced the average number of writhes, with an effect surpassing that of the standard drug diclofenac. The tail immersion method, on the other hand, is based on the principle that morphine-like substances can specifically prolong the latency of the tail withdrawal reflex in mice. The analgesic efficacy of the extract is assessed by the extension of the initial latency period [46]. The n-hexane fraction at both 200 and 400 mg/kg doses increased reaction time, % MPE (maximum possible effect), and latency elongation. This suggests the activation of a centrally mediated analgesic mechanism, as the extracts likely modulate central pain pathways [47]. Unlike the acetic acid test, this method minimizes the involvement of endogenous substances like prostaglandins, focusing instead on the sensitization of nociceptors by sensory nerves [48]. These findings indicate that the extracts may exert both peripheral and central analgesic effects. Many investigations reported that flavonoids, tannins, saponins, terpenoids, glycosides, and alkaloids contribute to the analgesic effect [49,50,51]. The presence of these phytochemicals in the plant extract could be responsible for a significant analgesic effect. Furthermore, GC-MS analysis of the plant extract revealed the presence of several bioactive compounds, including β-amyrone, phytol (3,7,11,15-tetramethyl-2-hexadecen-1-ol), lupeol, epilupeol, lup-20(29)-en-3-ol acetate, brasiliamide A, and stigmasterol. These phytoconstituents have been previously reported to exhibit significant analgesic activity, thereby supporting and reinforcing the outcomes of our experimental findings [52,53].

The findings of this thrombolytic research reveal that the coagulation process involves three key stages: prothrombin activator formation, thrombin generation, and fibrin production [54]. Antithrombotic or thrombolytic agents can interfere with these stages to prevent thrombus formation. The core mechanism of thrombolysis involves the elimination of fibrin through the activation of plasminogen into plasmin by inactive plasminogen activators. Streptokinase (SK) and urokinase (UK) function via an indirect clot lysis pathway, as confirmed by in vitro thrombolysis studies, where thrombolytic enzymes effectively degrade fibrin [55]. In this study, the methanolic extract of *D. bengalensis* demonstrated 33.38% blood clot lysis at a concentration of 20 mg/mL. The phytochemicals present in the extract may disrupt thrombus formation, thereby exhibiting thrombolytic activity. Previous research has elucidated the relative significance of alkaloids, saponins, tannins, terpenoids, polyphenols, coumarins, polysaccharides, lignans, lectins, proanthocyanidins, and flavonoids in their capacity as thrombolytic agents [18,23,24,56]. Our investigated extract’s phytochemical screening similarly confirms the existence of these bioactive compounds, thereby substantiating their role in contributing to the observed thrombolytic effect. Moreover, the GC-MS profile of the plant extract revealed the presence of γ-sitosterol, lupeol, phytol, vitamin E, and stigmasterol, which have been reported to contribute to thrombolytic action in different studies [57,58]. However, further advanced research is necessary to elucidate the exact mechanism of action and identify the specific bioactive compounds responsible for this effect. One limitation of this study is the lack of a priori power analysis. However, the observed effect sizes and statistically significant outcomes suggest the sample size was sufficient to detect relevant biological responses.

An essential method for evaluating the predicted ADME/T profiles of drugs whose activity against drug sites has not yet been measured is virtual screening. Compared to random screening, ADME evaluation can be used to more precisely identify potentially active chemicals based on their specified activity [59]. All of the compounds that were chosen for our ADME investigation complied with Lipinski’s rule of five, which ought to be considered when determining potential therapeutic targets.

PASS (Prediction of Activity Spectra for Substances) is a computational tool used to predict the biological activities of compounds based on their chemical structures. It evaluates the likelihood of bioactivity by calculating two key parameters: Pa (probability of activity) and Pi (probability of inactivity). Compounds with a higher Pa value than Pi are considered promising candidates for specific biological activities. In this research, compounds with Pa > Pi were deemed acceptable for further consideration regarding their biological potential [60]. The comprehensive analysis highlighted significant findings about the selected compounds, which could be attributed to the synergistic effects of multiple phytochemicals, including both well-established and previously unreported ones. This suggests that the combined action of these components may contribute to their observed bioactivity, offering new insights into their therapeutic potential.

Molecular docking studies are widely employed to predict ligand-target interactions and enhance our understanding of the bioactivities of natural products. These studies also provide insights into potential binding mechanisms within protein binding pockets [61]. In this research, molecular docking has clarified and supported the biological findings, offering a deeper comprehension of the observed effects. Using target proteins such as TNF-alpha (PDB: 2AZ5), human COX-2 inhibitor (PDB: 6COX), and tissue plasminogen activator, tPA (PDB: 1A5H), 23 selected compounds identified through GC-MS/MS analysis of ME, along with reference medications, were subjected to docking investigations. The goal was to predict potential biological modes of action, including anti-inflammatory, analgesic, and thrombolytic activities. The results revealed that the bioactive compounds of *D. bengalensis* exhibited excellent binding affinities (−8.6 to 6 kcal/mol) for the selected receptors. This strong binding affinity suggests that these compounds have a higher probability of being considered promising drug candidates due to their superior interactions with the target receptors. However, further investigation is recommended to determine whether these compounds demonstrate better receptor interactions compared to other bioactive components. Therefore, additional experimental validation is necessary to confirm the pharmacological effects of these isolated substances and to explore their therapeutic potential in greater detail.

## 4. Materials and Methods

### 4.1. Chemicals and Reagents

For analgesic and anti-inflammatory tests, diclofenac sodium (Square Pharmaceuticals Ltd., Dhaka, Bangladesh) served as the reference. Streptokinase was used as the thrombolytic standard (Renata Limited, Dhaka, Bangladesh). Merck supplied analytical-grade fractionation solvents, including n-hexane, ethyl acetate, and methanol. The remaining chemicals were all analytical grade and did not require any additional purification.

### 4.2. Plant Collection and Identification

For this current investigation, *Dischidia bengalensis Colebr* was collected from the Lovachora tea garden, 2 no. Lokkhi Prashad, West Union, Kanaighat, Sylhet, Bangladesh. The plant sample (specimen no: AI 020922-627) was identified by Dr. Shaikh Bokhtear Uddin, Professor, Department of Botany, University of Chittagong.

### 4.3. Extraction

After being cleaned, the leaves were allowed to dry and dried in the sunlight for seven days in a semi-shed in order to be ready for additional processing. They used a large capacity grinding machine to grind the leaves into a coarse powder. Five liters of methanol were used to soak 800 g of the powdered substance in a clean, round-bottomed flask. For 15 days, the container and its contents were kept together, shaken and stirred occasionally, and sealed with foil. Next, a new cotton plug was used to filter the entire mixture, and lastly, Whitman No. 1 filter paper. The filtrate was dried using a rotary evaporator (RE200, Bibby Sterling, Liverpool, UK). A limitation of the extraction methodology is the extended maceration time, which, while suitable for initial exploratory research, may raise concerns regarding economic viability for scale-up and the potential for enzymatic or microbial degradation despite the use of methanol as a solvent. Future work should focus on optimizing this process using modern, efficient techniques.

### 4.4. Solvent-Solvent Partition

After being suspended in distilled water, the dried methanolic crude extract underwent solvent-solvent partitioning using a modified Kupchan [62] method. To produce the corresponding solvent fractions, n-hexane, dichloromethane, and ethyl acetate were subjected to sequential fractionation in separatory funnels. Using a rotary evaporator, each solvent fraction was dried off under low pressure.

### 4.5. Experimental Design and Animals

The animals were all from Cumilla University’s animal research department in Bangladesh. The study used 120 male Swiss Albino mice that weighed between 25 and 28 g. The animals were housed in the laboratory animal room for at least a week before the investigation. Food and drink were served as needed, unless otherwise specified. For each test model, 50 mice in total were used. For each model, mice were divided into ten groups of five at random. Group I (1% tween 80 and DMSO in saline at dose 10 mL/kg, p.o.) was given a control; Group II received the standard drugs: indomethacin (10 mg/kg, p.o.) for the anti-inflammatory test, diclofenac sodium (10 mg/kg, p.o.) for the acetic acid-induced writhing test, and pentazocine (10 mg/kg, p.o.) for the tail immersion test; and Group III and IV were given various doses (200 and 400 mg/kg) of methanol extract; Group V and VI were given n-hexane; Group VI and VIII were given dichloromethane; and Group IX and X were given ethylacetate. Ethical approval was obtained from the Institutional Ethics Review Board (IERB), Eastern University, Dhaka, Bangladesh, under the approval no.- IERB-2024-0001, applied on 4 May 2024 and approved on 29 May 2024.

### 4.6. Acute Toxicity Evaluation and Dose Determination

Before the study, we conducted a pilot acute toxicity assessment following the procedure given in the paper by S. R. Afrin et al. [24] to confirm the safety limit of the extract. At first, 20 starved mice were divided into four groups of five. Then, each group received specific oral treatments, such as dosages of each extract ranging from 1 to 4 g/kg body weight. Following the oral injection of the extract, the mice were fasted for a further 3–4 h. After oral treatment with the extract, mice were monitored for alterations in their eyes, mucous membrane, skin, fur, circulatory rate, respiration rate, and autonomic and central nervous systems. These observations were made for the first 30 min following oral treatment, then every 24 h, with special attention paid to the first 4 h and then for 3 days to document any delayed toxicity. One-tenth of the median lethal dose (LD50) was taken as a therapeutic dose.

### 4.7. Phytochemical Screening

Phytochemical screening of the plant extract was conducted to qualitatively identify the presence of various bioactive constituents following standard procedures described by Harborne [63] and Trease and Evans [64].

### 4.8. Gas Chromatography-Mass Spectrometry (GC-MS) Analysis

The ME was analyzed using a Shimadzu GC–MS/MS TQ 8040 system with electron impact ionization. Separation was achieved on a fused silica capillary column (Rxi-30 m, 0.25 mm ID, 0.25 μm film thickness). Samples were injected at 250 °C, and the oven was programmed from 50 °C (1 min) to 200 °C (2 min) and 300 °C (7 min). The total run time was 40 min under 53.5 kPa pressure with a flow rate of 11 mL/min. The ion source temperature was 230 °C, detector voltage 0.5–0.6 kV, using Q3 scan mode (*m*/*z* 25–600), with a solvent cut time of 3.5 min. Compounds were tentatively identified by comparing their mass spectra to reference spectra inthe NIST and Wiley mass spectral libraries. Analyses were performed in Q3 scan mode (full scan), which provides a single dimension of spectral data. Future studies will aim to confirm these findings through the use of authentic chemical standards and the calculation of retention indices for greater confidence.

### 4.9. Anti-Inflammatory Activity

#### Carrageenan-Induced Paw Edema Test

Ten groups of five mice each, designated Group I through Group-X, were created from fifty randomly selected experimental animals. Mice were given free access to water and fasted for the whole night before the experiment. A control (1% tween 80 and DMSO in saline at dose 10 mL/kg, p.o.), standard (indomethacin 10 mg/kg), 200 mg/kg, and 400 mg/kg dose of *D. bengalensis* methanol extract (ME), dichloromethane soluble fraction, ethyl acetate soluble fraction, and n-hexane fraction were administered to each group, respectively. Before beginning any treatment, each mouse was precisely weighed, and the dosages of the test samples and control materials were modified accordingly. Mice were given carrageenan in the subplantar area of their left hind leg. Paw volume was then measured at 0, 1, 3, and 4 h following Nair et al.’s instructions [65]. Paw edema was expressed as the change in paw circumference (cm) by using the following formula:

(1) $$Inhibition\;of\;edema\;\left(\%\right)=\frac{\left(c_{t}-c_{o}\right)control-\left(c_{t}-c_{o}\right)treated}{\left(c_{t}-c_{o}\right)control}\times 100$$

Here, *C_t_* = Mean paw circumference for each group at different time intervals, *C_o_* = Mean paw circumference for each group before carrageenan injection.

### 4.10. Analgesic Activity

#### 4.10.1. Acetic Acid-Induced Writhing Method

This study was carried out using the method of Koster [66] as modified by Danbisya and Lee [67]. The treatment of mice was as outlined in the section on “Experimental design”. 0.7% acetic acid (10 mL/kg body weight) was injected intraperitoneally after 30 min of therapy. Beginning 15 min after the acetic acid injection, writing responses—which are typified by contractions of the abdominal muscles and elongation of the hind limbs—were recorded for 5 min [54]. The following ratio was used to determine the percentage of inhibition:

(2) $$\%\;of\;Inhibition=\;\frac{N_{c}-N_{t}}{N_{c}}\;\times 100$$

Here, *N_c_* = number of writhings in control, and *N_t_* = number of writhings in test animals.

#### 4.10.2. Tail Immersion

The central analgesic potential of the extracts was assessed in Swiss albino mice using the tail immersion method, adapted from the protocol of Kumar and Shankar (2009) [68]. A total of 50 fasted mice were randomly assigned to 10 groups, with 5 animals per group (*n* = 5). Group I served as the control and received a vehicle solution comprising 1% Tween-80 and DMSO in saline. Group II was treated with the reference analgesic pentazocine at a dose of 10 mg/kg. Groups III to X were administered either 200 or 400 mg/kg of the test samples PA, SPT, HA, or HV via oral gavage. All formulations, including the standard, were prepared in a suspension of Tween-80 and saline, vortexed to ensure uniform dispersion, and dosed according to each animal’s body weight.

To perform the test, 2–3 cm of each mouse’s tail was submerged in a water bath kept at a constant temperature of 50 ± 1 °C. Baseline (pre-treatment) tail withdrawal latencies were determined as the average of three measurements, each taken at 2 min intervals. Post-treatment latencies were recorded at 30, 60, 90, and 120 min using the same protocol. Mice showing baseline responses outside the range of 3–5 s were excluded from the analysis. A cut-off latency of 15 s was established to prevent thermal injury. The central analgesic response was quantified by calculating the percentage of the maximal possible effect (% *MPE*) using the formula:

(3) $$\%\;of\;MPE=\left\{\frac{\left(Postdrug\;latency-Predrug\;latency\right)}{\left(cut-off\;time\;(15.0\;s)-Predrug\;latency\right)}\right\}\times 100$$

### 4.11. Thrombolytic Activity

#### Blood Clot Lysis Method

All extracts’ thrombolytic activity was assessed using the Daginawala (2006) technique [18]. 20 mg/mL mother stock was prepared by dissolving 600 mg crude methanol extract into 30 mL NaCl solution (0.9%), which was further diluted to prepare concentrations of 10 mg/mL and 5 mg/mL. The lyophilized streptokinase (1,500,000 I.U.) vial (Renata Limited, Bangladesh) that is commercially available was utilized as the standard. After adding 5 mL of phosphate-buffered saline (PBS), everything was thoroughly mixed. The streptokinase concentration reached 30,000 I.U. Venous blood (500 μL/tube) from ten healthy participants was transferred to a separate pre-weighed sterile microcentrifuge tube and promptly citrated using a 3.1% sodium citrate solution. Each of these tubes was then filled with two hundred microliters of 2% calcium chloride, thoroughly mixed, and incubated for forty-five minutes at 37 °C to induce clotting. Each tube containing a clot was weighed once more to ascertain the clot weight after the serum was fully extracted (aspirated out without disrupting the clot that had formed). Then tubes were treated with 500 μL of positive control (standard), negative control, and the different concentrations of the extract, and incubated again at 45 °C for 90 min. Following clot lysis, the blood serum was extracted, and the tube was weighed once more to track any weight changes that coincided with the clot lysis progression [18,69]. The following formula was used to determine the clot lysis percentage:

(4) $$\%\;Clot\;Lysis=\;\frac{Weight\;of\;the\;lysis\;clot}{Weight\;of\;clot\;before\;lysis}\times \;100$$

### 4.12. In Silico Study

#### 4.12.1. Ligand Preparation

The 3D structures of 23 selected ME phytochemicals were retrieved from the PubChem database [70]. These substances were selected based on their documented biological actions, and they were then assessed further by contrasting them with accepted reference medications. PyRx was used for virtual screening to improve the possibility of finding active chemicals [71]. It made the format conversion to PDBQT and energy reduction easier. To rank the most promising options, the chosen compounds’ pharmacokinetic and drug-like characteristics were evaluated. ADMET (absorption, distribution, metabolism, excretion, and toxicity) characteristics were predicted using the pkCSM online platform (http://biosig.unimelb.edu.au/pkcsm/) [72] as of 31 July 2021. Additionally, Lipinski’s rule of five and other drug-likeness filters were applied through the Swiss ADME web server [73] to further refine the compound library and rule out poor candidates before docking studies.

#### 4.12.2. Protein Preparation

The RCSB Protein Data Bank provided the crystal structures of several target proteins, such as tissue plasminogen activator (PDB ID: 1A5H) for thrombolytic activity, human cyclooxygenase-2 inhibitor (COX-2; PDB ID: 6COX) for analgesic activity, and TNF-alpha (PDB ID: 2AZ5) for anti-inflammatory activity. These proteins’ active sites were determined using structural information that had already been published [74]. Protein preparation required several cleaning procedures utilizing Swiss-PdbViewer (v4.1) and BIOVIA Discovery Studio 4.5 Client, including the elimination of heteroatoms, co-crystallized ligands, and water molecules [75]. After that, polar hydrogen atoms were added, Kollman charges were assigned, and the MMFF94 force field was used to minimize energy in order to optimize the structures. To facilitate further docking and virtual screening investigations, the proteins were maintained in PDBQT format.

#### 4.12.3. Molecular Docking

Using a semi-flexible docking approach, Auto Dock Vina, integrated within the PyRx 0.8 platform, was used to dock the chosen protein–ligand complexes [76]. Before docking, the protein and ligand structures were energy-minimized and converted to PDBQT format using Auto Dock Tools [77]. Up to ten rotatable bonds were permitted to explore conformational space during docking, whereas the receptor proteins were considered as rigid and the ligand molecules retained full torsional flexibility. To provide adequate coverage of the binding pocket, the grid box was positioned in the middle of each target protein’s active site residues. The grid box’s dimensions and coordinates were established using Auto Dock Tools, guaranteeing that the whole active site area was covered. Using BIOVIA Discovery Studio Visualizer 2020, docking poses were visualized and assessed to examine important chemical interactions, binding affinities, and hydrogen bonding patterns.

### 4.13. Prediction of Activity Spectra for Substances (PASS)

The PASS online tools ‘Way2Drug–PASS Online, Available online: https://share.google/suNMcLtvn5Z8KHNxa (accessed on 11 September 2024)’ were used to investigate the PASS prediction and determine the likely biological effects of the chosen chemicals. Pa and Pi have values ranging from 0.000 to 1000. When a substance’s Pa value is higher than its Pi value, it is said to have biological potential. Pa < 0.5 indicates low pharmaceutical activity, Pa < 0.7 suggests moderate therapeutic potential, and Pa > 0.7 indicates strong medicinal activity [69].

### 4.14. Statistical Analysis

The standard error of the mean, or Mean± SEM, was used to present the findings. The statistical software “Statistical Package for the Social Sciences” (SPSS, Version 16.0, IBM Corporation, New York, NY, USA) was used to conduct the statistical analysis. Post hoc Dunnett test was used for comparisons following a one-way analysis of variance (ANOVA). The significance levels were determined using the following criteria: * *p* < 0.05, ** *p* < 0.01, and *** *p* < 0.001. When compared to the study group, these numbers demonstrate statistical significance. The phytochemical structures of the GC-MS compounds were drawn using ChemDraw Ultra 12.0.1, while MS Excel 2024 was utilized to illustrate the graphs. The sample size (*n* = 5 per group for in vivo studies and *n* = 10 for in vitrostudies) was chosen based on previous studies and standard practice in similar pharmacological evaluations. Although formal power analysis was not conducted, the sample size was considered adequate to detect meaningful differences.

## 5. Conclusions

The present study demonstrated that the methanolic extract and its multiple fractions (dichloromethane, N-hexane, and ethyl acetate) of *D. bengalensis* exhibited significant anti-inflammatory and analgesic activities. The crude methanolic extract also displayed moderate thrombolytic activity in the clot lysis test. Phytochemical screening and GC-MS analysis identified several secondary metabolites that may be responsible for these pharmacological effects. Furthermore, in silico molecular docking studies validated the experimental findings, supporting the observed biological activities. However, more advanced research is necessary to isolate and characterize the specific compounds responsible for these effects and to elucidate their underlying mechanisms of action. Such investigations could pave the way for the development of novel therapeutic agents from *D. bengalensis*.

## Figures and Tables

**Table 1:** Result of phytochemical test of methanolic extract (ME).

Serial No.	Tests for Phytoconstituents	Test Names	Inference
1	Alkaloids	(a) Wagner’s Test	+
		(b) Mayer’s Test	+
		(c) Dragendroff’s test	+
		(d) Hager’s test	-
2	Tannins	(a) Braymen’s Test	-
		(b) 10% sodium hydroxide test	+
3	Phytosterols	(a) Salkowski’s test	-
4	Terpenoids	(a) Chloroform and sulfuric acid Test	+
5	Flavonoids	(a) Zinc-hydrochloric acid reduction test	-
		(b) Lead acetate test	+
		(c) Alkaline reagent test	+
		(d) Conc. sulfuric acid test	+
		(e) 10% Ferric Chloride test	-
6	Saponins	(a) Sodium bicarbonate	-
		(b) Olive oil test	+
7	Glycosides	(a) Aqueous Sodium hydroxide test	+
8	Cardiac glycosides	(a) Keller-Killiani test	-
9	Quinones	(a) Conc. Hydrochloric acid test	-
10	Phenols	(a) 5%Ferric chloride test	+
		(b) Lead acetate test	+
		(c) Potassium dichromate test	+
11	Reducing sugars	(a) Fehling’s test	-
12	Carbohydrates	(a) Test for starch	+
13	Proteins and amino acids	(a) Xanthoproteic test	-
14	Carboxylic acids	(a) Effervescence test	-
15	Phlobatannins	(a) Hydrochloride test	-

Note: Presence (+), Absence (-).

**Table 2:** GC-MS compounds identified from the methanolic extract of *D. bengalensis*.

SL NO	Compound Name	R. Time	Area %
1	Hexanal	4.601	0.15
2	1-Dodecanol	11.89	0.10
3	Valeramide, 5-phenyl-*N*-methyl-	12.205	0.25
4	n-Pentadecanol	12.257	0.20
5	Decane, 1-iodo-	12.298	0.25
6	Phenol, 3,5-bis(1,1-dimethylethyl)-	12.535	0.20
7	(2E)-2-Heptylidenecyclohexanone	12.58	0.10
8	Phenol, 4-ethenyl-2,6-dimethoxy-	13.135	0.30
9	2-Bromotetradecane	13.35	0.32
10	.beta.-D-Mannofuranoside, 1-*O*-5-phenylpent-1-yl-	14.096	0.10
11	5-Octadecene, (*E*)-	14.22	0.23
12	Eicosane	14.422	0.30
13	Oxalic acid, cyclohexylmethyl tridecyl ester	14.496	0.13
14	Pentadecanal-	14.666	0.13
15	Methyl tetradecanoate	14.728	0.20
16	Pentadecanoic acid, methyl ester	16.04	0.20
17	Neophytadiene	16.204	0.62
18	2-Pentadecanone, 6,10,14-trimethyl-	16.297	1.28
19	3,7,11,15-Tetramethyl-2-hexadecen-1-ol	16.559	0.33
20	Calcitriol	16.709	0.18
21	9-methylheptadecane	17.125	0.10
22	Hexadecanoic acid, methyl ester	17.503	3.38
23	6-Pentadecenoic acid, 13-methyl-, (6*Z*)-	17.803	0.12
24	n-Hexadecanoic acid	18.078	12.99
25	Oxirane, [(hexadecyloxy)methyl]-	18.615	0.25
26	Hexadecanoic acid, 15-methyl-, methyl ester	19.078	0.18
27	Pentadecanoic acid	19.687	0.22
28	Cyclopentanol, 2,4,4-trimethyl-	20.009	0.13
29	9,12-Octadecadienoic acid (*Z*,*Z*)-, methyl ester	20.203	1.13
30	8,11,14-Docosatrienoic acid, methyl ester	20.308	1.17
31	11-Octadecenoic acid, methyl ester	20.4	0.15
32	Phytol	20.462	0.82
33	Methyl stearate	20.727	0.63
34	10*E*,12*Z*-Octadecadienoic acid	20.852	1.67
35	7-Tetradecenal, (*Z*)-	20.948	2.95
36	Tetracosanoic acid, isopropyl ester	21.2	0.18
37	Octadecanoic acid	21.328	2.18
38	Hexadecanamide	21.657	1.18
39	Benzoic acid, undecyl ester	22.104	0.17
40	Undec-10-ynoic acid, tetradecyl ester	22.949	0.38
41	9-Octadecynoic acid, methyl ester	23.661	0.12
42	4,8,12,16-Tetramethylheptadecan-4-olide	24.452	0.87
43	9,11-Octadecadienoic acid, methyl ester, (*E*,*E*)-	24.571	0.55
44	9-Octadecenamide, (*Z*)-	24.669	8.58
45	Octadecanamide	25.099	0.65
46	Eicosanal-	25.779	0.22
47	Hexanoic acid, heptadecyl ester	26.77	0.13
48	Hexadecanoic acid, 2-hydroxy-1-(hydroxymethyl)ethyl ester	27.133	0.48
49	Tricosanoic acid, methyl ester	28.912	0.15
50	Pentacosanal	28.997	0.13
51	Aciphyllene	29.465	0.17
52	4-Chlordehydromethyltestosterone	32.27	0.15
53	alpha-Tocospiro B	32.391	0.62
54	Lupeol	34.31	0.92
55	9,19-Cyclolanost-25-ene-3,24-diol	34.712	0.25
56	1-Phenanthrenecarboxylic acid, 7-ethyltetradecahydro-1,4a,7-trimethyl-, methyl ester, [1R-(1alpha,4abeta,4balpha,7beta)]	34.873	1.32
57	Cholesta-4,6-dien-3-ol, (3beta)-	35.322	2.55
58	Lup-20(29)-en-3-ol, acetate, (3beta)-	35.563	5.70
59	Epilupeol	35.959	0.18
60	Cholest-5-en-3-ol (3beta)-, carbonochloridate	36.1	0.33
61	Vitamin E	36.173	1.55
62	Lupeol, methyl ether	36.781	2.13
63	beta-Amyrone	37.019	4.00
64	Brasiliamide A, Me derivative	37.868	6.35
65	Stigmasterol	38.355	4.76
66	1,1,6-trimethyl-3-methylene-2-(3,6,9,13-tetramethyl-6-ethenye-10,14-dimethylene-pentadec-4-enyl)cyclohexane	38.47	0.90
67	17-(1,5-Dimethyl-3-phenylthiohex-4-enyl)-4,4,10,13,14-pentamethyl-2,3,4,5,6,7,10,11,12,13,14,15,16,17-tetradecahydro-1*H*-cyclope	38.776	4.30
68	(1*R*,4a*R*,5*S*)-5-[(*E*)-5-Hydroxy-3-methylpent-3-enyl]-1,4a-dimethyl-6-methylidene-3,4,5,7,8,8a-hexahydro-2*H*-naphthalene-1-carbaldeh	39.09	5.23
69	gamma-Sitosterol	39.603	8.20
70	24-Norursa-3,12-diene	39.841	2.97

**Table 3:** Screening of the anti-inflammatory effect of *D. bengalensis* and its different solvent fractions by calculating the mean paw circumference.

Sample	Pre-Injection Paw Circumference (cm)	Post-Injection Paw Circumference (cm) (% of Inhibition)			
		1st Hour	2nd Hour	3rd Hour	4th Hour
Control	0.98 ± 0.06	1.5 ± 0.04	1.5 ± 0.04	1.48 ± 0.04	1.5 ± 0.05
Indomethacin	1.02 ± 0.04	1.26 ± 0.05 ** (53.85)	1.24 ± 0.05 ** (57.69)	1.12 ± 0.04 *** (80)	1.04 ± 0.02 *** (96.15)
ME-400	1.26 ± 0.02	1.32 ± 0.04 (88.46)	1.3 ± 0.03 * (92.31)	1.3 ± 0.03 * (92.31)	1.3 ± 0.03 ** (92.31)
ME-200	1.26 ± 0.05	1.36 ± 0.05 (80.77)	1.34 ± 0.04 (84.62)	1.32 ± 0.04 * (88)	1.3 ± 0.03 ** (92.31)
NH-400	1.14 ± 0.02	1.32 ± 0.02 (65.38)	1.3 ± 0.03 * (69.23)	1.28 ± 0.02 ** (72)	1.22 ± 0.04 *** (84.62)
NH-200	1.18 ± 0.04	1.38 ± 0.04 (61.54)	1.36 ± 0.02 (65.38)	1.32 ± 0.02 * (72)	1.24 ± 0.02 *** (88.46)
DCM-400	1.16 ± 0.04	1.3 ± 0.07 * (73.08)	1.28 ± 0.07 ** (76.92)	1.26 ± 0.05 ** (80)	1.22 ± 0.04 *** (88.46)
DCM-200	1.18 ± 0.02	1.36 ± 0.05 (65.38)	1.34 ± 0.05 (69.2307)	1.32 ± 0.05 * (72)	1.24 ± 0.02 *** (88.46)
EA-400	1.14 ± 0.07	1.38 ± 0.06 (53.85)	1.36 ± 0.04 (57.69)	1.3 ± 0.04 * (68)	1.24 ± 0.05 ** (80.77)
EA-200	1.16 ± 0.05	1.4 ± 0.04 (53.85)	1.38 ± 0.04 (57.69)	1.36 ± 0.04 (60)	1.28 ± 0.04 (76.92)

All values are Mean ± SEM and statistically analyzed using One-Way Analysis of Variance (ANOVA) followed by Dunnett’s multiple comparison test, *n*  =  5. * *p* < 0.05, ** *p* < 0.01 and *** *p* < 0.001 were considered statistically significant as compared to control. ME = methanolic extract, DCM = dichloromethane fraction, NH = n-hexane fraction, EA = ethyl acetate fractions.

**Table 4:** Screening of peripheral analgesic activity of *D. bengalensis* and its different solvent fractions by the acetic acid-induced writhing method.

Group	Number of Writhing (Mean ± SEM)	% of Inhibition of Writhing
Control	46.9 ± 0.51	0
Diclofenac	5.6 ± 0.24 ***	88.06
ME-200	17.9 ± 0.33 ***	61.83
ME-400	7.8 ± 0.2 ***	83.37
NH-200	28.7 ± 0.58 **	38.81
NH-400	9.1 ± 0.24 ***	80.60
DCM-200	38.7 ± 0.2	17.48
DCM-400	21.5 ± 0.22 **	54.16
EA-200	16.2 ± 0.37 ***	65.46
EA-400	11.8 ± 0.37 ***	74.84

Note: All values are Mean ± SEM and statistically analyzed using One-Way Analysis of Variance (ANOVA) followed by Dunnett’s multiple comparison test, *n*  =  5. ** *p* < 0.01 and *** *p* < 0.001 were considered statistically significant as compared to control. ME = methanolic extract, DCM = dichloromethane fraction, NH = n-hexane fraction, EA = ethyl acetate fractions.

**Table 5:** Screening of central analgesic activity of *D. bengalensis* and its different solvent fractions by the tail immersion method.

Sample and Dose (mg/kg)	Reaction Times in Seconds (Mean ± SEM) and %MPE				
	Pre-Treatment	30 min	60 min	90 min	120 min
Control	3.5 ± 0.39	3.95 ± 0.14 (3.91)	3.44 ± 0.13 (−0.47)	3.87 ± 0.17 (3.23)	3.86 ± 0.11 *** (3.13)
Pentazocine-10	3.31 ± 0.47	7.73 ± 0.59 *** (37.73)	8.89 ± 0.59 *** (47.7)	10.23 ± 0.62 *** (59.19)	9.69 ± 0.45 *** (54.58)
ME-200	3.1 ± 0.15	9.05 ± 1.17 *** (50.03)	9.84 ± 1.17 *** (56.64)	9.29 ± 1.04 *** (52.03)	9.13 ± 1.17 *** (50.71)
ME-400	2.83 ± 0.30	7.26 ± 0.37 *** (36.39)	7.78 ± 0.37 *** (40.73)	9.09 ± 0.17 *** (51.42)	9.43 ± 0.69 *** (54.21)
NH-200	2.47 ± 0.35	7.34 ± 0.15 *** (38.89)	8.43 ± 0.15 *** (47.59)	9.03 ± 0.65 *** (52.36)	9.01 ± 0.22 *** (52.22)
NH-400	2.70 ± 0.42	7.83 ± 0.35 *** (41.69)	7.89 ± 0.35 *** (42.19)	9.29 ± 0.48 *** (53.56)	9.16 ± 0.19 *** (52.47)
EA-200	2.95 ± 0.41	7.15 ± 0.17 ** (34.87)	8.13 ± 0.17 *** (43.02)	8.82 ± 0.28 *** (48.73)	8.83 ± 0.26 *** (48.76)
EA-400	2.93 ± 0.34	7.45 ± 0.37 *** (37.46)	8.27 ± 0.37 *** (44.22)	8.76 ± 0.35 *** (48.28)	9.02 ± 0.32 *** (50.44)
DCM-200	3.46 ± 0.40	7.24 ± 0.28 ** (32.72)	8.30 ± 0.28 *** (41.96)	8.23 ± 0.33 *** (41.30)	8.73 ± 0.28 *** (45.70)
DCM-400	3.18 ± 0.26	7.36 ± 0.41 *** (35.42)	8.28 ± 0.41 *** (43.18)	8.48 ± 0.46 *** (44.89)	9.01 ± 0.33 *** (49.37)

Note: All values are Mean ± SEM and statistically analyzed using One-Way Analysis of Variance (ANOVA) followed by Dunnett’s multiple comparison test, *n*  =  5. ** *p* < 0.01 and *** *p* < 0.001 were considered statistically significant as compared to control. ME = methanolic extract, DCM = dichloromethane fraction, NH = n-hexane fraction, EA = ethyl acetate fraction.

**Table 6:** Percent elongation of latency time after administration of all test samples.

Sample	%Elongation of Latency Time			
	30 min	60 min	90 min	120 min
Control	-	-	-	-
Standard	48.93	61.28	62.20	60.22
ME-200	56.41	65.01	58.36	57.77
ME-400	45.62	55.78	57.43	59.09
NH-200	46.25	59.18	57.17	57.21
NH-400	49.60	56.39	58.36	57.89
EA-200	44.84	57.69	56.17	56.32
EA-400	47.06	58.38	55.84	57.25
DCM-200	45.47	58.54	52.98	55.85
DCM-400	46.41	58.44	54.41	57.22

Note: ME = methanolic extract, DCM = dichloromethane fraction, NH = n-hexane fraction, EA = ethyl acetate fraction.

**Table 7:** In silicoADMET analysis of reported phytochemicals of *Discidia bengalensis*.

Compounds Name	Absorption		Distribution		Metabolism	Excretion	Toxicity		Drug Likeliness	Bioavai- Lability
	Water Solubility (log mol/L)	Intestinal Absorption (Human) (%Absorbed)	VDss (Human) (log L/kg)	BBB Permeability (log BB)	CYP3A4 Substrate	Total Clearance (log mL/min/kg)	AMES Toxicity	Hepato- Toxicity		
Phenol, 3,5-bis(1,1-dimethylethyl)-	−4.972	94.942	0.047	0.297	No	0.733	No	No	-	-
Pentadecanoic acid, methyl ester	−5.874	95.372	0.508	0.772	No	1.629	No	No	Yes	0.55
Neophytadiene	−8.559	92.85	0.692	0.983	Yes	1.764	No	No	Yes	0.55
3,7,11,15-Tetramethyl-2-hexadecen-1-ol	−6.558	91.793	0.127	−0.16	Yes	1.798	No	No	Yes	0.55
Hexadecanoic acid, methyl ester	−6.927	92.335	0.334	0.749	Yes	1.861	No	No	Yes	0.55
n-Hexadecanoic acid	−5.562	92.004	−0.543	−0.111	Yes	1.763	No	No	Yes	0.85
9,12-Octadecadienoic acid (*Z*,*Z*)-, methyl ester	−7.343	92.66	0.272	0.767	Yes	2.032	No	No	-	-
8,11,14-Docosatrienoic acid, methyl ester	−7.902	91.792	0.152	0.832	Yes	2.211	No	No	Yes	0.55
11-Octadecenoic acid, methyl ester	−7.436	92.154	0.299	0.777	Yes	1.98	No	No	Yes	0.55
Phytol	−7.535	90.643	0.385	0.793	Yes	1.686	No	No	Yes	0.55
10E,12Z-Octadecadienoic acid	−5.862	92.329	−0.587	−0.142	Yes	1.931	No	Yes	-	-
9,11-Octadecadienoic acid, methyl ester, (*E*,*E*)-	−7.343	92.66	0.272	0.767	Yes	2.028	No	No	Yes	0.55
9-Octadecenamide, (*Z*)-	−7.074	90.218	0.281	−0.389	Yes	1.959	No	No	Yes	0.55
Aciphyllene	−5.547	96.209	0.687	0.767	No	1.217	No	No	Yes	0.55
Lupeol	−5.861	95.782	0	0.726	Yes	0.153	No	No	Yes	0.55
9,19-Cyclolanost-25-ene-3,24-diol	−5.626	93.699	−0.192	−0.357	Yes	0.344	No	Yes	Yes	0.55
Lup-20(29)-en-3-ol, acetate, (3beta)-	−6.006	97.894	−0.12	0.644	Yes	0.06	No	No	Yes	0.55
Epilupeol	−5.861	95.782	0	0.726	Yes	0.153	No	No	Yes	0.55
Lupeol, methyl ether	−6.144	97.862	0.043	0.832	Yes	0.18	No	No	Yes	0.55
beta-Amyrone	−6.674	97.473	0.224	0.693	Yes	−0.096	No	No	Yes	0.55
Stigmasterol	−6.682	94.97	0.178	0.771	Yes	0.618	No	No	Yes	0.55
gamma-Sitosterol	−6.773	94.464	0.193	0.193	Yes	0.628	No	No	Yes	0.55
24-Norursa-3,12-diene	−6.925	95.778	0.47	0.857	Yes	0.235	No	No	Yes	0.55

**Table 10:** Pass Prediction Analysis of Selected Bioactive Compounds of ME.

Compound Name	Biological Activity					
	Anti-Inflammatory		Analgesic		Thrombolytic	
	Pa	Pi	Pa	Pi	Pa	Pi
Phenol, 3,5-bis(1,1-dimethylethyl)-	0.610	0.029	0.489	0.043	0.283	0.013
Pentadecanoic acid, methyl ester	0.510	0.054	0.538	0.019	0.322	0.007
Neophytadiene	0.286	0.082	0.391	0.116	0.170	0.117
3,7,11,15-Tetramethyl-2-hexadecen-1-ol	0.300	0.182	0.458	0.070	0.346	0.005
Hexadecanoic acid, methyl ester	0.510	0.054	0.538	0.019	0.322	0.007
n-Hexadecanoic acid	0.515	0.052	0.526	0.023	0.326	0.006
9,12-Octadecadienoic acid (*Z*,*Z*)-, methyl ester	0.728	0.013	0.593	0.008	0.328	0.006
8,11,14-Docosatrienoic acid, methyl ester	0.728	0.013	0.593	0.008	0.328	0.006
11-Octadecenoic acid, methyl ester	0.607	0.030	0.573	0.011	0.353	0.005
Phytol	0.458	0.070	0.300	0.182	0.346	0.005
10E,12Z-Octadecadienoic acid	0.675	0.019	0.540	0.018	0.331	0.006
9,11-Octadecadienoic acid, methyl ester, (*E*,*E*)-	0.664	0.020	0.552	0.015	0.327	0.006
9-Octadecenamide, (*Z*)-	0.384	0.104	0.598	0.008	0.295	0.010
Aciphyllene	0.376	0.108	0.425	0.090	-	-
Lupeol	0.708	0.015	0.726	0.003	0.274	0.015
9,19-Cyclolanost-25-ene-3,24-diol	0.681	0.018	0.485	0.046	0.152	0.146
Lup-20(29)-en-3-ol, acetate, (beta)-	0.737	0.012	0.679	0.004	0.226	0.043
Epilupeol	0.708	0.015	0.726	0.003	0.274	0.015
Lupeol, methyl ether	0.713	0.014	0.641	0.005	0.231	0.039
beta-Amyrone	0.859	0.005	0.785	0.002	0.325	0.006
Stigmasterol	0.542	0.045	0.601	0.008	-	-
gamma-Sitosterol	0.467	0.067	0.558	0.014	-	-
24-Norursa-3,12-diene	0.900	0.004	0.606	0.007	0.219	0.050

## Data Availability

Upon reasonable request, the corresponding author will provide the data that backs up the study’s conclusions.

## Supplementary Materials

The following supporting information can be downloaded at https://www.mdpi.com/article/10.3390/molecules30183724/s1, Figure S1: GC-MS chromatogram of the methanolic extract of *D. bengalensis*.

## Abbreviations

The following abbreviations are used in this manuscript:

ME	methanolic extract
DCM	dichloromethane fraction
NH	n-hexane fraction
EA	ethyl acetate fractions
NSAIDs	nonsteroidal anti-inflammatory drugs
tPA	tissue plasminogen activator
GC-MS	gas chromatography-mass spectrometry
MPE	maximum possible effect
PASS	prediction of activity spectra for substances
