# Supplementary material for: Investigation of Analgesic, Anti-Inflammatory, and Thrombolytic Effects of Methanolic Extract and Its Fractions of Dischidia bengalensis: In Vitro and In Vivo Studies with In Silico Interventions
Source: Molecules. 2025 Sep 12;30(18):3724. doi: 10.3390/molecules30183724 (PMC12472498; doi:10.3390/molecules30183724)
Supplement: Supplementary file 1 [file molecules-30-03724-s001.zip › molecules-3831267-supplementary.pdf]

Supplementary Material

Investigation of analgesic, anti-inflammatory, and thrombolytic effects of methanolic extract and its fractions of *Dischidia bengalensis*: *In vitro* and *in vivo* studies with *in silico* interventions

Ainun Nahar [1,2], Md. Jahin Khandakar [3], Md. Jahirul Islam Mamun [1], Md. Hossain Rasel [1], Abu Bin Ihsan[2,*], Asef Raj [4], Saika Ahmed [5], Mohammed Kamrul Hossain [1], Md Riasat Hasan[6,*] and Takashi Saito[6,*]

[1] Department of Pharmacy, Faculty of Biological Sciences, University of Chittagong, Chittagong-4331, Bangladesh
[2] Department of Pharmacy, Faculty of Life Science, Eastern University, Dhaka-1345, Bangladesh
[3] Department of Oceanography, Faculty of Marine Science and Fisheries, University of Chittagong, Chittagong-4331, Bangladesh
[4] School of Pharmacy, BRAC University, Dhaka-1212, Bangladesh
[5] Department of Chemistry, University of Dhaka, Dhaka-1000, Bangladesh
[6] Division of Clinical Cariology and Endodontology, Department of Oral Rehabilitation, School of Dentistry, Health Sciences University of Hokkaido, Tobetsu 061-0293, Hokkaido, Japan
* Correspondence: ihsan.pharmacy@easternuni.edu.bd (A.B.I.); riasat@hoku-iryo-u.ac.jp (M.R.H.); t-saito@hoku-iryo-u.ac.jp (T.S.)

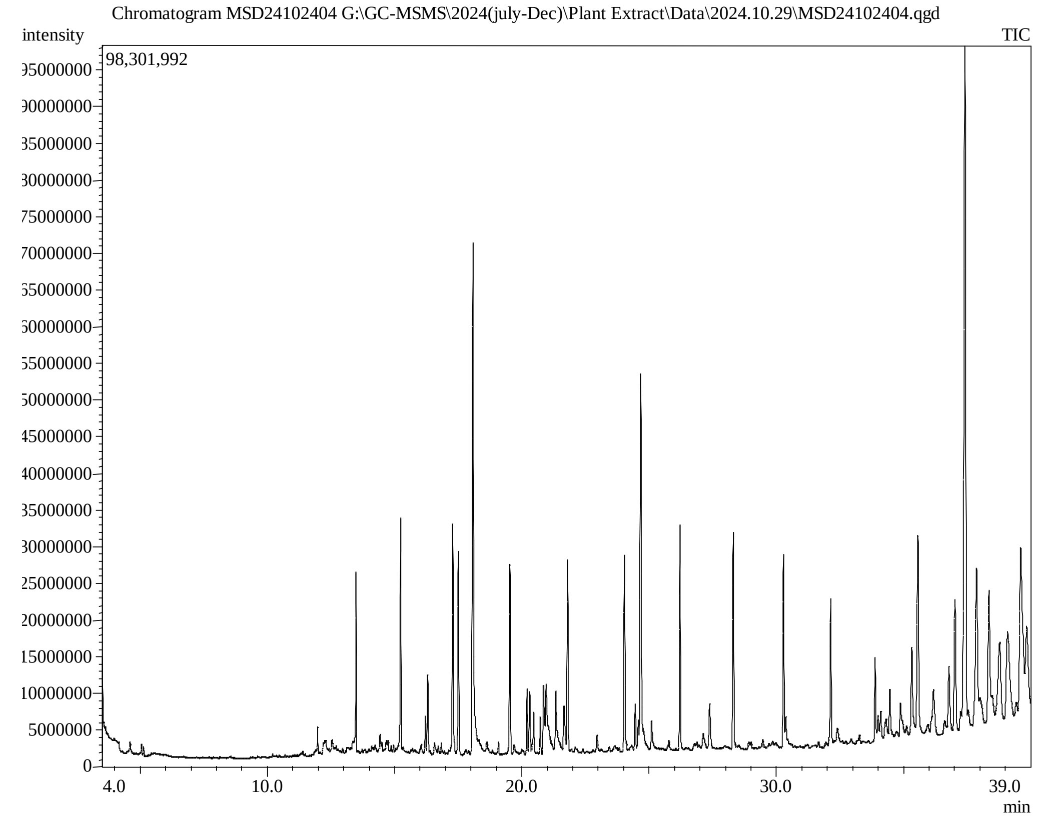

Figure S1. GC-MS chromatogram of the methanolic extract of *D. bengalensis*.
